# The Significance of a Discharge from the Ear

**Published:** 1905-03

**Authors:** 


### THE SIGNIFICANCE OF A DISCHARGE FROM THE EAR.

Edward B. Dench (*Medical Examiner and Practitioner*) says: In 1890 we find in the *British Medical Journal* a record of 9,000 consecutive autopsies held at Guy's Hospital between the years 1869 and 1888. Out of these 9,000 autopsies, in 57 cases death was due to aural suppuration, or one case in every 158 autopsies.

During twenty-one years at the Vienna General Hospital there were 40,073 autopsies; of these, 232 were due to aural suppuration, or one case in 173.

In the author's analysis of the New York Eye and Ear Infirmary Reports for eight years, he found that out of 64,858 aural cases treated in the institution, there had been 20 cases of cerebral abscess, 46 cases of sinus thrombosis, 24 cases of sinus thrombosis with ligation of the internal jugular, 119 cases of epidural abscess, 7 cases of cerebellar abscess and 2 cases of otitic meningitis, or 1 case in every 297 had a severe intracranial lesion. The number of serious intracranial complications was probably larger than this, as owing to a defect in our reports, only those cases of intracranial complication operated upon were recorded. During this period there were a number of deaths due to meningitis or other intracranial lesion, which were considered at the time inoperable, so that the number of intracranial lesions would really be much larger than this.

We can have no fact which shows more forcibly the importance of aural suppuration than that out of about 64,000 cases at an aural clinic, 1 case in every 297 should have suffered from a severe intracranial lesion. Going into the author's statistics more thoroughly, we find that out of the 64,858 cases treated at the clinic 4,836 were suffering from an acute purulent middle-ear inflammation, while 14,847 were suffering from a chronic purulent inflammation. In other words, there were 19,323 cases, of which 14,487 were suffering from a chronic purulent inflammation; there were 19,323 cases which suffered from a discharge from the ear, and of this number, 218 cases, or 1 case in every 88, suffered from some severe intracranial complication. Owing to the imperfect condition of the hospital reports it is impossible to tell in what proportion of the cases suffering from intracranial complications the severe lesion was the result of a chronic discharge from the ear; and in no cases was it due simply to an acute condition. However, acute purulent inflammations of the middle-ear are only rarely followed by any secondary lesion within the cranium. On the other hand, the lighting up of an acute process within an ear which has been the seat of a previous chronic suppurative inflammation, is exceedingly prone to be followed by some severe intracranial lesion.

The findings at the time of operation on these cases show almost invariably that while the disease has been considered dormant, and while it has given rise to no symptoms, it has steadily progressed, the erosion of bone has continued until either the dura is exposed in the middle or posterior cranial fossa, or until actual infection of the brain-substance itself has occurred. Even after the intracranial structures are involved no serious symptoms may follow until an acute process is suddenly grafted upon the chronic one; then symptoms of intracranial involvement rapidly supervene, and at this time it often happens that surgical interference, although promptly instituted, comes too late to save the life of the patient.—*Medical Review of Reviews.*

